# 1,3-Bis(2-anilino-2-oxoeth­yl)-1*H*-imidazol-3-ium chloride acetonitrile monosolvate

**DOI:** 10.1107/S1600536811048410

**Published:** 2011-11-19

**Authors:** Chuang-Yi Liao, Hon Man Lee

**Affiliations:** a National Changhua University of Education, Department of Chemistry, Changhua, Taiwan 50058

## Abstract

In the title compound, C_19_H_19_N_4_O_2_^+^·Cl^−^·C_2_H_3_N, the dihedral angle between the two phenyl rings is 69.57 (8)° while the dihedral angles between the imidazole ring and the phenyl rings are 70.61 (7) and 82.11 (7)°. In the crystal, N—H⋯Cl, C—H⋯O, C—H⋯Cl and C—H⋯N hydrogen bonds link the imidazolium cations, chloride anions and acetonitrile solvent mol­ecules into a two-dimensional hydrogen-bonded network parallel to (001); an intra­molecular C—H⋯O hydrogen bond is also observed.

## Related literature

For the crystal structures of nickel, palladium, and silver complexes with ligands derived from the title compound, see: Liao, Chan, Chang *et al.* (2007 ▶), Liao, Chan, Zeng *et al.* (2007 ▶) and Liao *et al.* (2008 ▶), respectively.
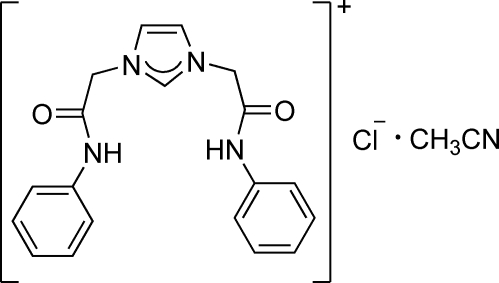


## Experimental

### 

#### Crystal data

C_19_H_19_N_4_O_2_^+^·Cl^−^·C_2_H_3_N
*M* *_r_* = 411.89
Triclinic, 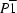

*a* = 8.7801 (6) Å
*b* = 10.4544 (6) Å
*c* = 12.1998 (7) Å
α = 91.842 (4)°
β = 95.492 (4)°
γ = 108.096 (4)°
*V* = 1057.28 (11) Å^3^
*Z* = 2
Mo *K*α radiation
μ = 0.21 mm^−1^
*T* = 150 K
0.22 × 0.20 × 0.15 mm

#### Data collection

Bruker SMART APEXII diffractometer
Absorption correction: multi-scan (*SADABS*; Sheldrick, 2003 ▶) *T*_min_ = 0.956, *T*_max_ = 0.970
13815 measured reflections
5067 independent reflections
3597 reflections with *I* > 2σ(*I*)
*R*_int_ = 0.040

#### Refinement

*R*[*F* ^2^ > 2σ(*F* ^2^)] = 0.048
*wR*(*F* ^2^) = 0.129
*S* = 1.06
5067 reflections
257 parameters
H-atom parameters constrained
Δρ_max_ = 0.96 e Å^−3^
Δρ_min_ = −0.90 e Å^−3^

### 

Data collection: *APEX2* (Bruker, 2007 ▶); cell refinement: *SAINT* (Bruker, 2007 ▶); data reduction: *SAINT*; program(s) used to solve structure: *SHELXTL* (Sheldrick, 2008 ▶); program(s) used to refine structure: *SHELXTL*; molecular graphics: *SHELXTL*; software used to prepare material for publication: *DIAMOND* (Brandenburg, 2006 ▶).

## Supplementary Material

Crystal structure: contains datablock(s) I, global. DOI: 10.1107/S1600536811048410/wn2459sup1.cif

Structure factors: contains datablock(s) I. DOI: 10.1107/S1600536811048410/wn2459Isup2.hkl

Supplementary material file. DOI: 10.1107/S1600536811048410/wn2459Isup3.cml

Additional supplementary materials:  crystallographic information; 3D view; checkCIF report

## Figures and Tables

**Table 1:** Hydrogen-bond geometry (Å, °)

*D*—H⋯*A*	*D*—H	H⋯*A*	*D*⋯*A*	*D*—H⋯*A*
N3—H3*A*⋯Cl1^i^	0.88	2.28	3.1624 (17)	175
N4—H4⋯Cl1^ii^	0.88	2.35	3.2287 (17)	175
C4—H4*B*⋯N5	0.99	2.50	3.262 (3)	134
C12—H12*A*⋯O1^iii^	0.99	2.25	3.126 (2)	147
C12—H12*B*⋯Cl1^iii^	0.99	2.67	3.400 (2)	131
C19—H19⋯O2	0.95	2.31	2.911 (2)	121
C20—H20*A*⋯O2	0.98	2.32	3.238 (3)	156

Symmetry codes: (i) 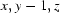; (ii) 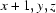; (iii) 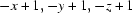.

**Fig. 1.**
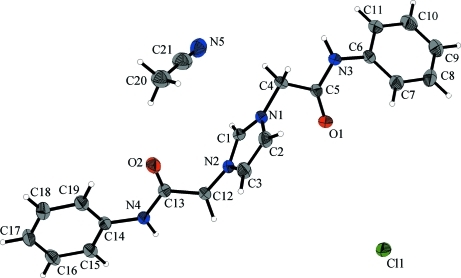
The structure of the title compound, showing 50% probability displacement ellipsoids for the non-hydrogen atoms. The H atoms are shown as spheres of arbitrary radius.

**Fig. 2.**
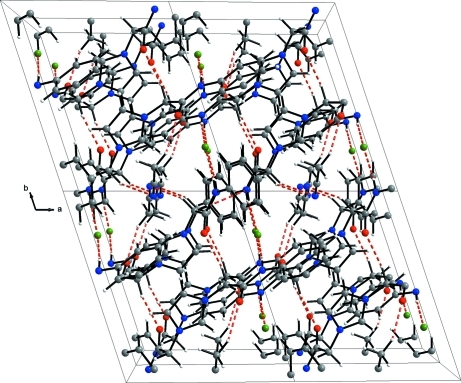
A view of the crystal packing along the c axis, displaying the hydrogen bonds as dashed lines.

## supplementary crystallographic information

### Comment

The title compound, C_19_H_19_N_4_O^+^.Cl^-^.C_2_H_3_N, is a precursor for N-heterocyclic carbene (NHC) ligands. Nickel (Liao, Chan, Chang *et al.,* 2007), palladium (Liao, Chan, Zeng *et al.,* 2007) and silver (Liao *et al.,* 2008) complexes with NHC ligands derived from the salt have been reported by us previously.

The crystal structure of the title compound is shown in Fig. 1. The dihedral angle between the two phenyl rings is 69.57 (8)°; the dihedral angles between the imidazole ring and the phenyl rings are 70.61 (7)° for [C6–C11] and 82.11 (7)° for [C14–C19].

After deprotonation and metal coordination via C2, the N—C—N bond angles become slightly smaller. The N—C—N bond angle in the title compound is 108.59 (17)°, whereas the angles are 105.5 (2) and 106.3 (3) ° in the palladium complex (Liao, Chan, Zeng *et al.*, 2007), 104.4 (4)° in the silver complex (Liao *et al.*, 2008) and 104.9 (4)° in the nickel complex (Liao, Chan, Chang *et al.*, 2007).

One non-classical intramolecular C—H···O hydrogen bond has been detected (Table 1), whereas classical and non-classical intermolecular hydrogen bonds of the type N—H···Cl, C—H···Cl, C—H···O and C—H···N link the imidazolium cations, chloride anions and the acetonitrile molecules into a two-dimensional hydrogen-bonded network (Fig. 2).

### Experimental

The compound was prepared according to the literature procedure (Liao, Chan, Zeng *et al.,* 2007). Suitable crystals were obtained by slow diffusion of diethyl ether into an acetonitrile solution of the compound at room temperature.

### Refinement

All H atoms could have been detected in the difference Fourier map; nevertheless, all H atoms were positioned geometrically and refined as riding atoms, with Csp^2^—H = 0.95, C(methyl)—H = 0.98, C(methylene)—H = 0.99, and N—H = 0.88 Å; *U*_iso_(H) = x*U*_eq_(C,N), where x = 1.5 for methyl H and 1.2 for all other H atoms.

### Crystal data

**Table:**

C_19_H_19_N_4_O_2_^+^·Cl^−^·C_2_H_3_N	*Z* = 2
*M**_r_* = 411.89	*F*(000) = 432
Triclinic, *P*1	*D*_x_ = 1.294 Mg m^−^^3^
Hall symbol: -P 1	Mo *K*α radiation, λ = 0.71073 Å
*a* = 8.7801 (6) Å	Cell parameters from 2360 reflections
*b* = 10.4544 (6) Å	θ = 2.5–23.9°
*c* = 12.1998 (7) Å	µ = 0.21 mm^−^^1^
α = 91.842 (4)°	*T* = 150 K
β = 95.492 (4)°	Block, white
γ = 108.096 (4)°	0.22 × 0.20 × 0.15 mm
*V* = 1057.28 (11) Å^3^	

### Data collection

**Table:**

Bruker SMART APEXII diffractometer	5067 independent reflections
Radiation source: fine-focus sealed tube	3597 reflections with *I* > 2σ(*I*)
graphite	*R*_int_ = 0.040
ω scans	θ_max_ = 28.0°, θ_min_ = 1.7°
Absorption correction: multi-scan (*SADABS*; Sheldrick, 2003)	*h* = −11→11
*T*_min_ = 0.956, *T*_max_ = 0.970	*k* = −13→13
13815 measured reflections	*l* = −16→16

### Refinement

**Table:**

Refinement on *F*^2^	Primary atom site location: structure-invariant direct methods
Least-squares matrix: full	Secondary atom site location: difference Fourier map
*R*[*F*^2^ > 2σ(*F*^2^)] = 0.048	Hydrogen site location: inferred from neighbouring sites
*wR*(*F*^2^) = 0.129	H-atom parameters constrained
*S* = 1.06	*w* = 1/[σ^2^(*F*_o_^2^) + (0.0538*P*)^2^ + 0.377*P*] where *P* = (*F*_o_^2^ + 2*F*_c_^2^)/3
5067 reflections	(Δ/σ)_max_ = 0.001
257 parameters	Δρ_max_ = 0.96 e Å^−^^3^
0 restraints	Δρ_min_ = −0.90 e Å^−^^3^

### Special details

**Table:**

Geometry. All e.s.d.'s (except the e.s.d. in the dihedral angle between two l.s. planes) are estimated using the full covariance matrix. The cell e.s.d.'s are taken into account individually in the estimation of e.s.d.'s in distances, angles and torsion angles; correlations between e.s.d.'s in cell parameters are only used when they are defined by crystal symmetry. An approximate (isotropic) treatment of cell e.s.d.'s is used for estimating e.s.d.'s involving l.s. planes.
Refinement. Refinement of *F*^2^ against ALL reflections. The weighted *R*-factor *wR* and goodness of fit *S* are based on *F*^2^, conventional *R*-factors *R* are based on *F*, with *F* set to zero for negative *F*^2^. The threshold expression of *F*^2^ > σ(*F*^2^) is used only for calculating *R*-factors(gt) *etc*. and is not relevant to the choice of reflections for refinement. *R*-factors based on *F*^2^ are statistically about twice as large as those based on *F*, and *R*- factors based on ALL data will be even larger.

### Fractional atomic coordinates and isotropic or equivalent isotropic displacement parameters (Å^2^)

**Table:**

	*x*	*y*	*z*	*U*_iso_*/*U*_eq_	
C1	0.5447 (2)	0.28079 (19)	0.54543 (15)	0.0241 (4)	
H1	0.5994	0.2311	0.5071	0.029*	
C2	0.3554 (2)	0.3390 (2)	0.62012 (16)	0.0305 (4)	
H2	0.2536	0.3360	0.6423	0.037*	
C3	0.4941 (2)	0.4426 (2)	0.64010 (17)	0.0310 (5)	
H3	0.5086	0.5259	0.6798	0.037*	
C4	0.2737 (2)	0.11100 (19)	0.51452 (16)	0.0275 (4)	
H4A	0.1824	0.0846	0.5598	0.033*	
H4B	0.3256	0.0394	0.5162	0.033*	
C5	0.2105 (2)	0.12423 (19)	0.39559 (15)	0.0247 (4)	
C6	0.0091 (2)	−0.0133 (2)	0.24692 (16)	0.0261 (4)	
C7	−0.0460 (3)	0.0873 (2)	0.20240 (17)	0.0356 (5)	
H7	−0.0171	0.1739	0.2400	0.043*	
C8	−0.1437 (3)	0.0597 (2)	0.10267 (19)	0.0429 (6)	
H8	−0.1814	0.1282	0.0721	0.052*	
C9	−0.1872 (3)	−0.0659 (2)	0.04710 (19)	0.0440 (6)	
H9	−0.2535	−0.0836	−0.0214	0.053*	
C10	−0.1329 (3)	−0.1656 (2)	0.09242 (19)	0.0431 (6)	
H10	−0.1628	−0.2523	0.0549	0.052*	
C11	−0.0351 (2)	−0.1401 (2)	0.19235 (18)	0.0336 (5)	
H11	0.0013	−0.2092	0.2231	0.040*	
C12	0.7777 (2)	0.4883 (2)	0.59194 (16)	0.0266 (4)	
H12A	0.7821	0.5827	0.5810	0.032*	
H12B	0.8238	0.4563	0.5296	0.032*	
C13	0.8785 (2)	0.4833 (2)	0.69954 (16)	0.0261 (4)	
C14	1.1599 (2)	0.59148 (19)	0.78694 (15)	0.0255 (4)	
C15	1.3085 (2)	0.6761 (2)	0.76428 (17)	0.0305 (4)	
H15	1.3176	0.7179	0.6961	0.037*	
C16	1.4440 (2)	0.7002 (2)	0.84055 (18)	0.0350 (5)	
H16	1.5457	0.7569	0.8240	0.042*	
C17	1.4306 (3)	0.6414 (2)	0.94059 (18)	0.0380 (5)	
H17	1.5229	0.6573	0.9929	0.046*	
C18	1.2824 (3)	0.5596 (2)	0.96405 (18)	0.0400 (5)	
H18	1.2734	0.5204	1.0333	0.048*	
C19	1.1455 (2)	0.5332 (2)	0.88779 (17)	0.0335 (5)	
H19	1.0442	0.4762	0.9046	0.040*	
C20	0.5812 (3)	0.1257 (3)	0.8373 (2)	0.0472 (4)	
H20A	0.6785	0.2017	0.8313	0.071*	
H20B	0.6011	0.0734	0.8992	0.071*	
H20C	0.4918	0.1598	0.8499	0.071*	
C21	0.5404 (3)	0.0403 (3)	0.7362 (2)	0.0472 (4)	
Cl1	0.07301 (6)	0.75633 (5)	0.49516 (4)	0.03031 (14)	
N1	0.38999 (17)	0.23796 (16)	0.56116 (12)	0.0244 (3)	
N2	0.61030 (17)	0.40496 (15)	0.59229 (12)	0.0238 (3)	
N3	0.10210 (18)	0.00739 (16)	0.35129 (13)	0.0265 (4)	
H3A	0.0878	−0.0630	0.3914	0.032*	
N4	1.02870 (18)	0.57136 (16)	0.70405 (13)	0.0263 (4)	
H4	1.0477	0.6231	0.6482	0.032*	
N5	0.5089 (2)	−0.0260 (2)	0.65673 (17)	0.0439 (5)	
O1	0.25525 (16)	0.22902 (13)	0.34914 (11)	0.0291 (3)	
O2	0.82600 (17)	0.40675 (16)	0.77037 (12)	0.0387 (4)	

### Atomic displacement parameters (Å^2^)

**Table:**

	*U*^11^	*U*^22^	*U*^33^	*U*^12^	*U*^13^	*U*^23^
C1	0.0221 (9)	0.0205 (9)	0.0291 (9)	0.0059 (7)	0.0011 (7)	0.0047 (7)
C2	0.0240 (9)	0.0343 (12)	0.0340 (10)	0.0116 (9)	0.0005 (8)	−0.0007 (9)
C3	0.0265 (10)	0.0311 (11)	0.0363 (11)	0.0121 (9)	−0.0005 (8)	−0.0058 (9)
C4	0.0208 (9)	0.0232 (10)	0.0328 (10)	−0.0004 (8)	−0.0012 (7)	0.0044 (8)
C5	0.0168 (8)	0.0237 (10)	0.0329 (10)	0.0055 (7)	0.0011 (7)	0.0022 (8)
C6	0.0183 (8)	0.0273 (10)	0.0304 (10)	0.0042 (8)	0.0008 (7)	0.0031 (8)
C7	0.0351 (11)	0.0306 (12)	0.0392 (11)	0.0118 (9)	−0.0074 (9)	−0.0044 (9)
C8	0.0421 (13)	0.0409 (13)	0.0448 (13)	0.0168 (11)	−0.0129 (10)	0.0025 (10)
C9	0.0399 (13)	0.0430 (14)	0.0411 (12)	0.0076 (11)	−0.0135 (10)	−0.0044 (10)
C10	0.0414 (13)	0.0334 (13)	0.0459 (13)	0.0040 (10)	−0.0075 (10)	−0.0097 (10)
C11	0.0294 (10)	0.0250 (11)	0.0423 (12)	0.0043 (8)	−0.0016 (9)	0.0014 (9)
C12	0.0206 (9)	0.0229 (10)	0.0326 (10)	0.0027 (7)	−0.0024 (7)	0.0042 (8)
C13	0.0224 (9)	0.0241 (10)	0.0302 (10)	0.0061 (8)	−0.0004 (7)	0.0027 (8)
C14	0.0232 (9)	0.0236 (10)	0.0279 (9)	0.0066 (8)	−0.0023 (7)	−0.0008 (8)
C15	0.0269 (10)	0.0291 (11)	0.0323 (10)	0.0048 (8)	0.0010 (8)	0.0010 (8)
C16	0.0223 (10)	0.0343 (12)	0.0431 (12)	0.0033 (9)	−0.0011 (8)	−0.0045 (9)
C17	0.0305 (11)	0.0399 (13)	0.0392 (12)	0.0102 (9)	−0.0124 (9)	−0.0039 (10)
C18	0.0387 (12)	0.0432 (13)	0.0334 (11)	0.0096 (10)	−0.0074 (9)	0.0041 (10)
C19	0.0278 (10)	0.0337 (12)	0.0337 (11)	0.0035 (9)	−0.0016 (8)	0.0047 (9)
C20	0.0476 (10)	0.0426 (10)	0.0472 (10)	0.0069 (8)	0.0084 (8)	0.0076 (8)
C21	0.0476 (10)	0.0426 (10)	0.0472 (10)	0.0069 (8)	0.0084 (8)	0.0076 (8)
Cl1	0.0255 (2)	0.0263 (3)	0.0385 (3)	0.00651 (19)	0.00336 (18)	0.0089 (2)
N1	0.0181 (7)	0.0238 (9)	0.0290 (8)	0.0039 (6)	−0.0009 (6)	0.0038 (6)
N2	0.0186 (7)	0.0217 (8)	0.0298 (8)	0.0061 (6)	−0.0024 (6)	0.0019 (6)
N3	0.0228 (8)	0.0211 (8)	0.0323 (8)	0.0031 (7)	−0.0015 (6)	0.0048 (7)
N4	0.0217 (8)	0.0252 (9)	0.0282 (8)	0.0027 (7)	−0.0015 (6)	0.0071 (7)
N5	0.0382 (11)	0.0497 (13)	0.0481 (12)	0.0195 (9)	0.0047 (9)	0.0088 (10)
O1	0.0266 (7)	0.0229 (7)	0.0345 (7)	0.0035 (6)	0.0012 (6)	0.0061 (6)
O2	0.0291 (8)	0.0409 (9)	0.0363 (8)	−0.0024 (7)	−0.0021 (6)	0.0141 (7)

### Geometric parameters (Å, °)

**Table:**

C1—N1	1.327 (2)	C12—N2	1.458 (2)
C1—N2	1.330 (2)	C12—C13	1.523 (2)
C1—H1	0.9500	C12—H12A	0.9900
C2—C3	1.350 (3)	C12—H12B	0.9900
C2—N1	1.385 (2)	C13—O2	1.221 (2)
C2—H2	0.9500	C13—N4	1.349 (2)
C3—N2	1.373 (2)	C14—C15	1.389 (3)
C3—H3	0.9500	C14—C19	1.390 (3)
C4—N1	1.458 (2)	C14—N4	1.416 (2)
C4—C5	1.529 (3)	C15—C16	1.390 (3)
C4—H4A	0.9900	C15—H15	0.9500
C4—H4B	0.9900	C16—C17	1.383 (3)
C5—O1	1.221 (2)	C16—H16	0.9500
C5—N3	1.352 (2)	C17—C18	1.379 (3)
C6—C11	1.388 (3)	C17—H17	0.9500
C6—C7	1.392 (3)	C18—C19	1.396 (3)
C6—N3	1.417 (2)	C18—H18	0.9500
C7—C8	1.387 (3)	C19—H19	0.9500
C7—H7	0.9500	C20—C21	1.446 (3)
C8—C9	1.383 (3)	C20—H20A	0.9800
C8—H8	0.9500	C20—H20B	0.9800
C9—C10	1.384 (3)	C20—H20C	0.9800
C9—H9	0.9500	C21—N5	1.133 (3)
C10—C11	1.390 (3)	N3—H3A	0.8800
C10—H10	0.9500	N4—H4	0.8800
C11—H11	0.9500		
			
N1—C1—N2	108.59 (17)	H12A—C12—H12B	108.0
N1—C1—H1	125.7	O2—C13—N4	126.00 (17)
N2—C1—H1	125.7	O2—C13—C12	122.57 (17)
C3—C2—N1	106.85 (17)	N4—C13—C12	111.43 (17)
C3—C2—H2	126.6	C15—C14—C19	119.79 (17)
N1—C2—H2	126.6	C15—C14—N4	116.74 (17)
C2—C3—N2	107.19 (17)	C19—C14—N4	123.47 (18)
C2—C3—H3	126.4	C14—C15—C16	120.5 (2)
N2—C3—H3	126.4	C14—C15—H15	119.7
N1—C4—C5	110.81 (15)	C16—C15—H15	119.7
N1—C4—H4A	109.5	C17—C16—C15	119.9 (2)
C5—C4—H4A	109.5	C17—C16—H16	120.1
N1—C4—H4B	109.5	C15—C16—H16	120.1
C5—C4—H4B	109.5	C18—C17—C16	119.65 (18)
H4A—C4—H4B	108.1	C18—C17—H17	120.2
O1—C5—N3	125.89 (17)	C16—C17—H17	120.2
O1—C5—C4	122.59 (16)	C17—C18—C19	121.2 (2)
N3—C5—C4	111.52 (16)	C17—C18—H18	119.4
C11—C6—C7	120.09 (17)	C19—C18—H18	119.4
C11—C6—N3	118.26 (17)	C14—C19—C18	119.0 (2)
C7—C6—N3	121.52 (17)	C14—C19—H19	120.5
C8—C7—C6	119.33 (19)	C18—C19—H19	120.5
C8—C7—H7	120.3	C21—C20—H20A	109.5
C6—C7—H7	120.3	C21—C20—H20B	109.5
C9—C8—C7	121.0 (2)	H20A—C20—H20B	109.5
C9—C8—H8	119.5	C21—C20—H20C	109.5
C7—C8—H8	119.5	H20A—C20—H20C	109.5
C8—C9—C10	119.3 (2)	H20B—C20—H20C	109.5
C8—C9—H9	120.4	N5—C21—C20	179.6 (3)
C10—C9—H9	120.4	C1—N1—C2	108.53 (16)
C9—C10—C11	120.6 (2)	C1—N1—C4	125.20 (16)
C9—C10—H10	119.7	C2—N1—C4	126.02 (16)
C11—C10—H10	119.7	C1—N2—C3	108.84 (16)
C6—C11—C10	119.7 (2)	C1—N2—C12	125.67 (16)
C6—C11—H11	120.2	C3—N2—C12	125.49 (16)
C10—C11—H11	120.2	C5—N3—C6	126.67 (17)
N2—C12—C13	111.46 (16)	C5—N3—H3A	116.7
N2—C12—H12A	109.3	C6—N3—H3A	116.7
C13—C12—H12A	109.3	C13—N4—C14	128.50 (17)
N2—C12—H12B	109.3	C13—N4—H4	115.8
C13—C12—H12B	109.3	C14—N4—H4	115.8

### Hydrogen-bond geometry (Å, °)

**Table:**

*D*—H···*A*	*D*—H	H···*A*	*D*···*A*	*D*—H···*A*
N3—H3A···Cl1^i^	0.88	2.28	3.1624 (17)	175.
N4—H4···Cl1^ii^	0.88	2.35	3.2287 (17)	175.
C4—H4B···N5	0.99	2.50	3.262 (3)	134.
C12—H12A···O1^iii^	0.99	2.25	3.126 (2)	147.
C12—H12B···Cl1^iii^	0.99	2.67	3.400 (2)	131.
C19—H19···O2	0.95	2.31	2.911 (2)	121.
C20—H20A···O2	0.98	2.32	3.238 (3)	156.

Symmetry codes: (i) *x*, *y*−1, *z*; (ii) *x*+1, *y*, *z*; (iii) −*x*+1, −*y*+1, −*z*+1.
